# Supplementary material for: Loss avoidance during social interactions
Source: Commun Psychol. 2025 Jul 24;3:114. doi: 10.1038/s44271-025-00288-5 (PMC12289979; doi:10.1038/s44271-025-00288-5)
Supplement: Supplementary file 2 — Supplementary Information [file 44271_2025_288_MOESM2_ESM.pdf]

Supplementary information for Kuper-Smith & Korn, Loss Avoidance During Social Interactions, Communications Psychology, 2025

This PDF includes:

Figs. S1 to S3
Tables S1 to S8

Data and code availability:

All data collected for this study can be found at: https://github.com/dnhi-lab/losses_gains_2x2 and archived on Zenodo: https://zenodo.org/records/15667770.

All code used for the simulations, analyses, and figures mentioned in this manuscript can be found at: https://github.com/dnhi-lab/losses_gains_2x2 and archived on Zenodo: https://zenodo.org/records/15667770.

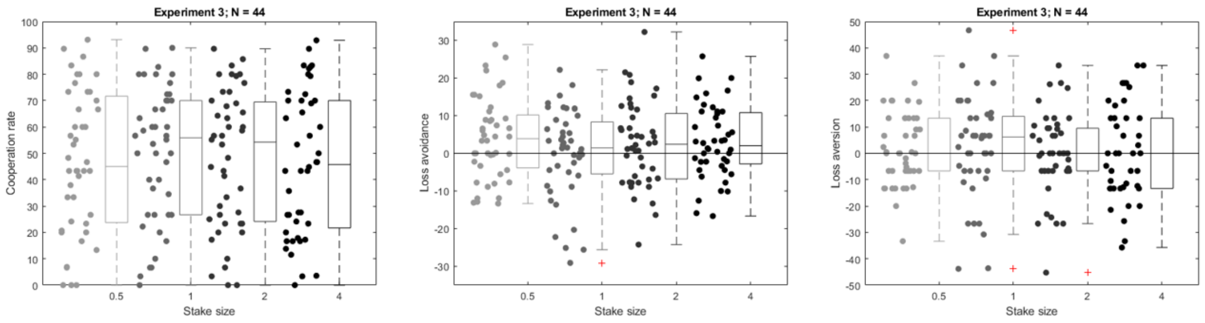

Fig. S1.

The effect of stake size on cooperation rate (left), loss avoidance (centre) and loss aversion (right) in Experiment 3. Neither effect (of stake size on cooperation, loss avoidance, and loss aversion) is significant. For loss aversion, the median loss aversion score is exactly 0 for stake sizes 0.5, 2, and 4, such that the horizontal line covers the median values in this figure. The box plots display the median, the interquartile range, and 1.5 * the interquartile range.

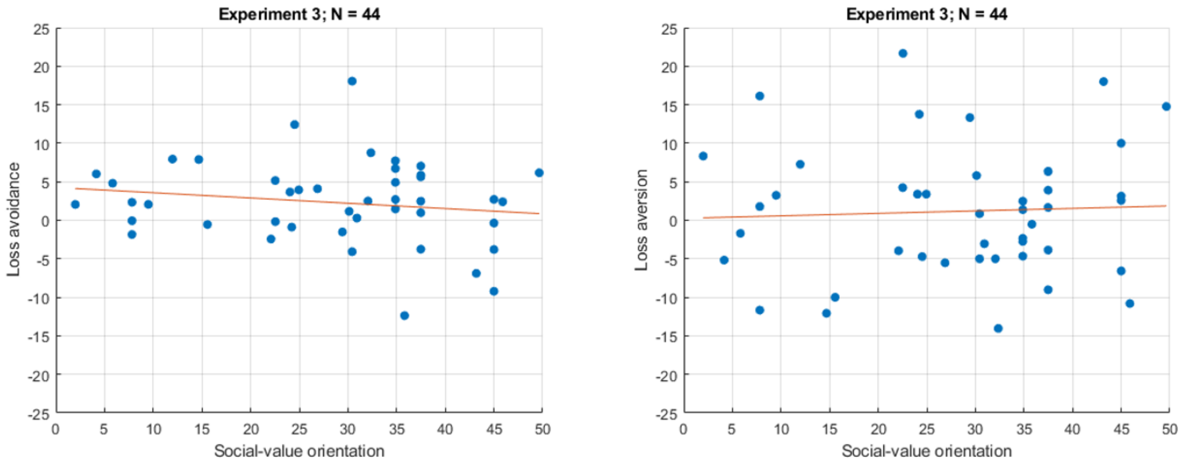

Fig. S2.
The effect of social-value orientation on loss avoidance (left) and loss aversion (right) per participant in Experiment 3. Neither regression is significant.

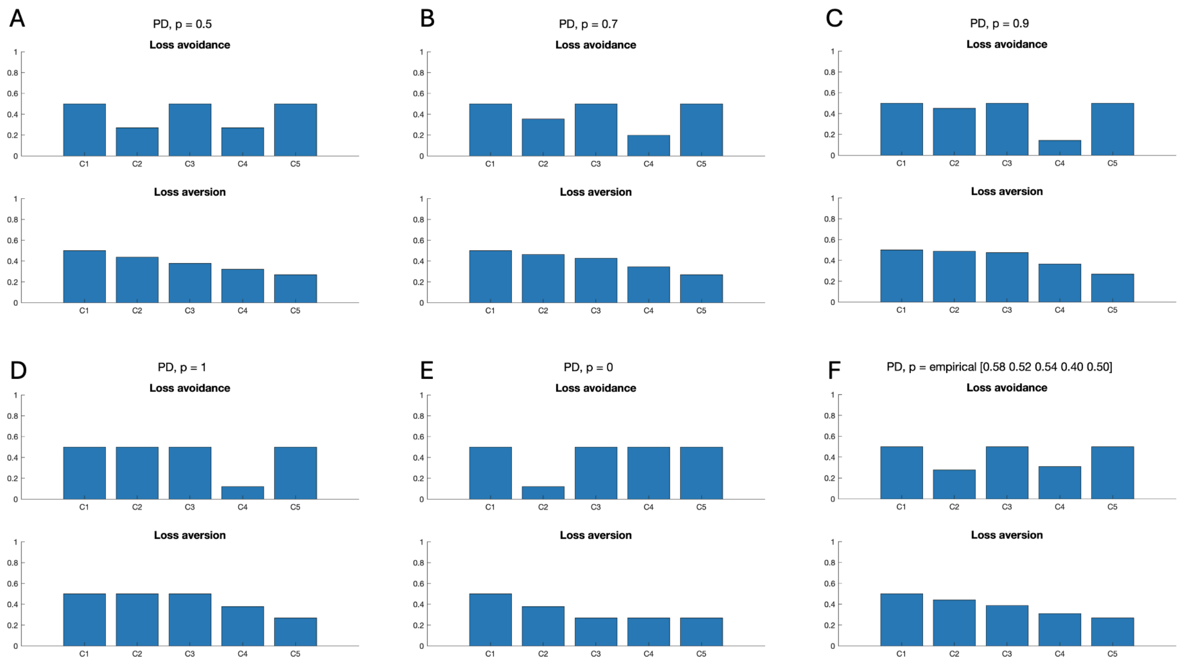

Fig. S3.

How different probabilities of what the other person will do affect the predicted cooperation rates. These examples use the Prisoner's Dilemma, but the same principles apply to Stag Hunt and Chicken, too. A) The predicted cooperation rates for loss avoidance and loss aversion, assuming a 50% chance the other player will cooperate. B), C), and D) show how the cooperation rates change as the expectation that the other will cooperate go from 50% to 100%. This shift leads to a shift in where the effect happens (e.g., for loss avoidance, with 50% expectation, the effect is of equal magnitude for C2 and C4; but with certainty that the other will cooperate, the effect exists only for C4, but much larger). E) shows the predictions with certainty that the other will defect, with a reverse pattern observed to the pattern show in D. These shifts show that under extreme cases certain hypotheses can break down: our hypotheses are about potential gain-loss asymmetries, so some hypotheses fall apart if losses or gains become increasingly (un)likely. For example, in C2, if a player knows the other will cooperate, the only negative outcome is no longer possible (because the other player will not defect). F) In Experiment 7, we asked participants to rate the probability that the other person would cooperate. The mean responses were close to 50% (range: 40%-58%). If we use the mean expected cooperation rate of the other player (separately for each category), then the predictions are practically indistinguishable from the 50% simplification.

Experiment 1 (Prisoner's Dilemma)							
Category	Payoff matrices T	R	P	S	Base POM	*a	+b
C1	7	5	3	2	1	1	+4
	6	4	2	1	1	1	+3
C2	5	3	1	0	1	1	+2
	4.5	2.5	0.5	-0.5	1	1	+1.5
	4	2	0	-1	1	1	+1
C3	3.5	1.5	-0.5	-1.5	1	1	+0.5
	3	1	-1	-2	1	1	0
	2	0	-2	-3	1	1	-1
C4	1	-1	-3	-4	1	1	-2
	0	-2	-4	-5	1	1	-3
C5	-1	-3	-5	-6	1	1	-4
	-2	-4	-6	-7	1	1	-5
Experiment 2 (Prisoner's Dilemma)							
Category	Payoff matrices T	R	P	S	Base POM	*a	+b
C1	15	11	7	3	2	1	+9
	13	9	5	1	2	1	+7
	28	20	14	6	3	1	+17
	26	18	12	4	3	1	+15
C2	11	7	3	-1	2	1	+5
	9	5	1	-3	2	1	+3
	20	12	6	-2	3	1	+9
	18	10	4	-4	3	1	+7
C3	7	3	-1	-5	2	1	+1
	5	1	-3	-7	2	1	-1
	12	4	-2	-10	3	1	+1
	10	2	-4	-12	3	1	-1
C4	3	-1	-5	-9	2	1	-3
	1	-3	-7	-11	2	1	-5
	4	-4	-10	-18	3	1	-7
	2	-6	-12	-20	3	1	-9
C5	-1	-5	-9	-13	2	1	-7
	-3	-7	-11	-15	2	1	-9
	-4	-12	-18	-26	3	1	-15
	-6	-14	-20	-28	3	1	-17
Experiment 3 (Prisoner's Dilemma)							
Category	Payoff matrices T	R	P	S	Base POM	*a	+b
C1	7	5	3	1	2	0.5	+8
	14	10	6	2	2	1	+8
	28	20	12	4	2	2	+8
	56	40	24	8	2	4	+8
C2	5	3	1	-1	2	0.5	+4
	10	6	2	-2	2	1	+4
	20	12	4	-4	2	2	+4
	40	24	8	-8	2	4	+4
C3	3	1	-1	-3	2	0.5	0
	6	2	-2	-6	2	1	0
	12	4	-4	-12	2	2	0
	24	8	-8	-24	2	4	0
C4	1	-1	-3	-5	2	0.5	-4
	2	-2	-6	-10	2	1	-4

	4	-4	-12	-20	2	2	-4
	8	-8	-24	-40	2	4	-4
C5	-1	-3	-5	-7	2	0.5	-8
	-2	-6	-10	-14	2	1	-8
	-4	-12	-20	-28	2	2	-8
	-8	-24	-40	-56	2	4	-8
Experiment 4 (Prisoner's Dilemma)							
Category	Payoff matrices				Base	*a	+b
	T	R	P	S	POM		
C1	56	40	24	8	2	4	+8
C2	40	24	8	-8	2	4	+4
C3	24	8	-8	-24	2	4	0
C4	8	-8	-24	-40	2	4	-4
C5	-8	-24	-40	-56	2	4	-8
Experiment 5 (Prisoner's Dilemma)							
Category	Payoff matrices				Base	*a	+b
	T	R	P	S	POM		
C1	560	400	240	80	2	40	+8
C2	400	240	80	-80	2	40	+4
C3	240	80	-80	-240	2	40	0
C4	80	-80	-240	-400	2	40	-4
C5	-80	-240	-400	-560	2	40	-8
Experiment 6							
Category	Payoff matrices				Base	*a	+b
	T	R	P	S	POM		
C1 PD	560	400	240	80	2	40	+8
SH	400	560	240	80	2	40	+8
C2 PD	400	240	80	-80	2	40	+4
SH	240	400	80	-80	2	40	+4
C3 PD	240	80	-80	-240	2	40	0
SH	80	240	-80	-240	2	40	0
C4 PD	80	-80	-240	-400	2	40	-4
SH	-80	80	-240	-400	2	40	-4
C5 PD	-80	-240	-400	-560	2	40	-8
SH	-240	-80	-400	-560	2	40	-8
Experiment 7							
Category	Payoff matrices				Base	*a	+b
	T	R	P	S	POM		
C1 PD	560	400	240	80	2	40	+8
SH	400	560	240	80	2	40	+8
CH	560	400	80	240	2	40	+8
C2 PD	400	240	80	-80	2	40	+4
SH	240	400	80	-80	2	40	+4
CH	400	240	-80	80	2	40	+4
C3 PD	240	80	-80	-240	2	40	0
SH	80	240	-80	-240	2	40	0
CH	240	80	-240	-80	2	40	0
C4 PD	80	-80	-240	-400	2	40	-4
SH	-80	80	-240	-400	2	40	-4
CH	80	-80	-400	-240	2	40	-4
C5 PD	-80	-240	-400	-560	2	40	-8
SH	-240	-80	-400	-560	2	40	-8
CH	-80	-240	-560	-400	2	40	-8

Table S1.

All payoff matrices used in the 7 experiments. The payoff matrices were created by taking what we call a base-payoff-matrix, which was allways C3, and then adding changing +b to create the five categories. This way, there is a symmetry across the 5 categories, such that e.g., for the Prisoner's Dilemma the average payoff of Categories 2 and 4 is the same as the average payoff of the Categories 1, 3, and 5, such that when comparing for loss avoidance in the Prisoner's Dilemma, there is no difference in average payoff across that contrast. In Experiment 1, a constant (+b) was added/subtracted from each outcomes such that each of the possible 4 outcomes (T, R, P, S) was positive, 0, and negative at some point. This was changed for the later experiments: we no longer included payoffs with 0, because that was difficult to interpret with respect to loss aversion and loss avoidance (because some payoff matrices include some positive, a neutral, and some negative outcomes), whereas having outcomes as either positive or negative always leads to payoff matrices that can be categorized unambigously. Multiplying a payoff matrix with *a leads to different levels of stake size (e.g., in Experiment 3, we have four levels of stake size, ranging from 0.5 to 4), without changing the category of the payoff matrix. C1-C5 = Category 1 to Category 5, PD = Prisoner's Dilemma, SH = Stag Hunt, CH = Chicken.

Predictor	Statistic	Model 1	Model 2	Model 3
Loss avoidance	Estimate	-0.096	-0.132	-0.151
	Standard error	0.026	0.030	0.035
	z-value	-3.648	-4.434	-4.358
	p-value	< 0.001	< 0.001	< 0.001
Loss aversion (linear fit of Category)	Estimate		0.012	0.038
	Standard error		0.030	0.034
	z-value		0.402	1.120
	p-value		0.687	0.263
Previous decision other	Estimate			2.495
	Standard error			0.032
	z-value			78.646
	p-value			< 0.001
Intercept	Estimate	-0.122	-0.109	-1.339
	Standard error	0.151	0.151	0.135
	z-value	-0.810	-0.724	-9.914
	p-value	0.418	0.469	< 0.001
Model	AIC	35771	35768	28350

Table S2.

The results from the mixed effects models of the combined data for Experiments 1-3. P-values in red indicate significant predictors at the p = 0.05 level.

Predictor	Statistic	Model PD1	Model PD2
Loss avoidance	Estimate	0.577	0.487
	Standard error	0.199	0.224
	t-value	2.900	2.174
	p-value	0.004	0.030
Loss aversion	Estimate	0.146	0.100
	Standard error	0.069	0.076
	t-value	2.120	1.321
	p-value	0.034	0.187
Expectation	Estimate		-0.035
	Standard error		0.004
	t-value		-8.647
	p-value		< 0.001
Intercept	Estimate	-0.403	1.234
	Standard error	0.238	0.321
	t-value	-1.690	3.842
	p-value	0.091	< 0.001
Model	AIC	605	514
	Chi-squared	13.1	106
	p-value	0.002	< 0.001

Table S3.

Results from the logistic regressions for Experiment 5. P-values in red indicate significant predictors at the p = 0.05 level.

Predictor	Statistic	Model SH1	Model SH2	Model SH3
Loss avoidance	Estimate	1.187	1.197	2.350
	Standard error	0.228	0.283	0.660
	t-value	5.215	4.224	3.559
	p-value	< 0.001	< 0.001	< 0.001
Loss aversion	Estimate	-0.638	-0.419	-0.561
	Standard error	0.203	0.227	0.436
	t-value	-3.143	-1.844	-1.289
	p-value	0.002	0.065	0.200
Expectation	Estimate		-0.052	-0.052
	Standard error		0.005	0.013
	t-value		-9.813	-4.000
	p-value		< 0.001	< 0.001
Loss avoidance : expectation	Estimate			-0.021
	Standard error			0.010
	t-value			-2.055
	p-value			0.040
Loss aversion : expectation	Estimate			0.003
	Standard error			0.008
	t-value			0.335
	p-value			0.738
Intercept	Estimate	-0.983	1.740	1.755
	Standard error	0.333	0.454	0.724
	t-value	-2.953	3.835	2.425
	p-value	0.003	< 0.001	0.015
Model	AIC	442	305	304
	Chi-squared	39.7	179	184
	p-value	< 0.001	< 0.001	< 0.001

Table S4.
All preregistered logistic regressions for Stag Hunt for Experiment 6. P-values in red indicate significant predictors at the p = 0.05 level.

Predictor	Statistic	Model PD1	Model PD2	Model PD3
Loss avoidance	Estimate	1.112	0.818	1.119
	Standard error	0.173	0.120	0.409
	t-value	6.415	4.175	2.736
	p-value	< 0.001	< 0.001	0.006
Loss aversion	Estimate	0.245	0.157	0.026
	Standard error	0.063	0.069	0.144
	t-value	3.917	2.272	0.183
	p-value	< 0.001	0.023	0.855
Expectation	Estimate		-0.039	-0.045
	Standard error		0.004	0.011
	t-value		-9.961	-4.319
	p-value		< 0.001	< 0.001
Loss avoidance : expectation	Estimate			-0.007
	Standard error			0.008
	t-value			-0.793
	p-value			0.428
Loss aversion : expectation	Estimate			0.003
	Standard error			0.003
	t-value			0.996
	p-value			0.319
Intercept	Estimate	-1.382	0.830	1.096
	Standard error	0.226	0.313	0.529
	t-value	-6.106	2.651	2.073
	p-value	< 0.001	0.008	0.038
Model	AIC	779	652	654
	Chi-squared	58.3	188	189
	p-value	< 0.001	< 0.001	< 0.001

Table S5.
All preregistered logistic regressions for the Prisoner's Dilemma for **Experiment 6**. P-values in red indicate significant predictors at the $p = 0.05$ level.

Predictor	Statistic	Model CH1	Model CH2	Model CH3
Loss avoidance	Estimate	1.203	1.005	0.936
	Standard error	0.167	0.170	0.329
	t-value	7.211	5.904	2.843
	p-value	< 0.001	< 0.001	0.005
Loss aversion	Estimate	0.154	0.122	0.006
	Standard error	0.132	0.137	0.303
	t-value	1.168	0.887	0.019
	p-value	0.243	0.375	0.985
Expectation	Estimate		-0.022	-0.027
	Standard error		0.004	0.013
	t-value		-5.645	-2.050
	p-value		< 0.001	0.040
Loss avoidance : expectation	Estimate			0.001
	Standard error			0.006
	t-value			0.225
	p-value			0.822
Loss aversion : expectation	Estimate			0.002
	Standard error			0.005
	t-value			0.425
	p-value			0.671
Intercept	Estimate	-1.493	-0.321	-0.038
	Standard error	0.320	0.384	0.742
	t-value	-4.667	-0.835	-0.051
	p-value	< 0.001	0.404	0.959
Model	AIC	647	615	619
	Chi-squared	60.6	94.4	94.6
	p-value	< 0.001	< 0.001	< 0.001

Table S6.
All preregistered logistic regressions for Chicken for **Experiment 7**. P-values in red indicate significant predictors at the $p = 0.05$ level.

Predictor	Statistic	Model SH1	Model SH2	Model SH3
Loss avoidance	Estimate	0.449	0.440	0.753
	Standard error	0.173	0.198	0.357
	t-value	2.602	2.228	2.109
	p-value	0.009	0.026	0.035
Loss aversion	Estimate	-0.413	-0.456	-0.792
	Standard error	0.152	0.182	0.367
	t-value	-2.728	-2.496	-2.156
	p-value	0.006	0.013	0.031
Expectation	Estimate		-0.042	-0.053
	Standard error		0.004	0.011
	t-value		-10.457	-4.782
	p-value		< 0.001	< 0.001
Loss avoidance : expectation	Estimate			-0.006
	Standard error			0.006
	t-value			-1.073
	p-value			0.283
Loss aversion : expectation	Estimate			0.006
	Standard error			0.006
	t-value			1.080
	p-value			0.280
Intercept	Estimate	-0.785	1.900	2.519
	Standard error	0.272	0.405	0.707
	t-value	-2.889	4.688	3.564
	p-value	0.004	< 0.001	< 0.001
Model	AIC	570	430	432
	Chi-squared	14.4	156	158
	p-value	< 0.001	< 0.001	< 0.001

Table S7.

All preregistered logistic regressions for Stag Hunt for **Experiment 7**. P-values in red indicate significant predictors at the p = 0.05 level.

Predictor	Statistic	Model PD1	Model PD2	Model PD3
Loss avoidance	Estimate	0.644	0.480	0.042
	Standard error	0.169	0.186	0.405
	t-value	3.800	2.582	0.104
	p-value	< 0.001	0.010	0.917
Loss aversion	Estimate	0.193	0.133	0.058
	Standard error	0.060	0.066	0.148
	t-value	3.208	2.020	0.396
	p-value	0.001	0.043	0.692
Expectation	Estimate		-0.035	-0.044
	Standard error		0.004	0.010
	t-value		-9.195	-4.442
	p-value		< 0.001	< 0.001
Loss avoidance : expectation	Estimate			0.009
	Standard error			0.008
	t-value			1.231
	p-value			0.218
Loss aversion : expectation	Estimate			0.002
	Standard error			0.003
	t-value			0.596
	p-value			0.551
Intercept	Estimate	-1.125	0.837	1.272
	Standard error	0.216	0.305	0.533
	t-value	-5.204	2.742	2.386
	p-value	< 0.001	0.006	0.017
Model	AIC	805	704	706
	Chi-squared	25.5	129	131
	p-value	< 0.001	< 0.001	< 0.001

Table S8.

All preregistered logistic regressions for the Prisoner's Dilemma for **Experiment 7**. P-values in red indicate significant predictors at the $p = 0.05$ level.
